# Comparative Genomics and Characterization of *Shigella flexneri* Isolated from Urban Wastewater

**DOI:** 10.1264/jsme2.ME23105

**Published:** 2024-06-05

**Authors:** Sarmishta Mukhopadhyay, Meesha Singh, Mahashweta Mitra Ghosh, Santanu Chakrabarti, Sayak Ganguli

**Affiliations:** 1 Post Graduate and Research Department of Biotechnology, St. Xavier’s College (Autonomous) Kolkata, West Bengal, India; 2 Post Graduate and Research Department of Microbiology, St. Xavier’s College (Autonomous) Kolkata, West Bengal, India; 3 Department of Zoology, Government General Degree College Singur, Hooghly, West Bengal, India

**Keywords:** *Shigella flexneri*, WGS ana­lysis, comparative pan-genomics, antimicrobial resistance

## Abstract

*Shigella* species are a group of highly transmissible Gram-negative pathogens. Increasing reports of infection with extensively drug-resistant varieties of this stomach bug has convinced the World Health Organization to prioritize *Shigella* for novel therapeutic interventions. We herein coupled the whole-genome sequencing of a natural isolate of *Shigella flexneri* with a pangenome ana­lysis to characterize pathogen genomics within this species, which will provide us with an insight into its existing genomic diversity and highlight the root causes behind the emergence of quick vaccine escape variants. The isolated novel strain of *S. flexneri* contained ~4,500 protein-coding genes, 57 of which imparted resistance to antibiotics. A comparative pan-genomic ana­lysis revealed genomic variability of ~64%, the shared conservation of core genes in central metabolic processes, and the enrichment of unique/accessory genes in virulence and defense mechanisms that contributed to much of the observed antimicrobial resistance (AMR). A pathway ana­lysis of the core genome mapped 22 genes to 2 antimicrobial resistance pathways, with the bulk coding for multidrug efflux pumps and two component regulatory systems that are considered to work synergistically towards the development of resistance phenotypes. The prospective evolvability of *Shigella* species as witnessed by the marked difference in genomic content, the strain-specific essentiality of unique/accessory genes, and the inclusion of a potent resistance mechanism within the core genome, strengthens the possibility of novel serotypes emerging in the near future and emphasizes the importance of tracking down genomic diversity in drug/vaccine design and AMR governance.

Antimicrobial resistance (AMR) exemplified by quickly evolving bacterial strains, outcompeting modern medicines, is no longer a myth, but a grim reality, accounting for 1.27 million deaths in 2019 alone. With several comprehensive global studies forecasting AMR causing 10 million deaths yearly by 2050, the World Health Organization has endorsed the dire need for a synchronized surveillance strategy to monitor the emergence, transmission, trends, and burden of‍ ‍AMR across the globe (Antimicrobial Resistance Collaborators, 2022). Recent studies that traced the origin and dissemination of resistant bacteria highlighted the importance of environmental reservoirs as hotspots, fostering enrichment and exchange within the antibiotic resistance gene pool (Mukherjee *et al.*, 2021). Environmental basins receiving hospital effluents with a large mass of residual antibiotics and resistant bacteria are considered to exert a positive selection pressure for the survival and spread of resistant bacteria (Triggiano *et al.*, 2020). Therefore, tracking AMR dynamics in environmental hubs has become imperative to gather epidemiological data on resistant pathogens and implement policies to contain their spread. Prompt advances in whole-genome sequencing (WGS) technologies and its applications have contributed to the real-time detection of factors contributing to AMR. While the suitability of phenotypic assays in AMR surveillance remains controversial, the vast amount of structural data generated by WGS helps to infer current trends more logically and with greater precision based on the presence/absence of specific mole­cular markers, the co-occurrence of specific genes, and their transmission potential depending on their genetic context (association with mobile genetic elements [MGEs]) (Hendriksen *et al.*, 2019). The gradual shift in the paradigm of antibiotic resistance mechanisms from modifying enzymes and efflux pumps to quickly transposable genetic elements has accelerated the acquisition of resistance in some microbial families, such as Enterobacteriaceae (Iredell *et al.*, 2016).

We herein performed a WGS ana­lysis of a multidrug-resistant (MDR) strain of *Shigella flexneri* isolated from the main drainage system in Purulia district of West Bengal, India. The present study aimed to characterize the resistance genes and associated antimicrobial phenotype of the environmental isolate and provide a comparative profile of the genetic content between the isolated strain and 15 other *S. flexneri* strains, typically selected from geographical zones that have significant vulnerability to shigellosis. This research focused on both contemporaneous and non-coeval comparisons between a naturally isolated MDR strain of *S. flexneri* and 15 other *S. flexneri* strains reportedly collected from different Asian countries, and has the potential to highlight existing diversity and identify genomic factors that promote the convergent evolution of resistance to standard antimicrobials. The present results, which integrate a comparative genomic ana­lysis with a resistance gene investigation, provide a framework for the optimization of treatment strategies and the selection of target candidates for drug design.

The choice of *Shigella* was based on the growing and unchecked pervasiveness of infections (700,000 cases annually worldwide), with 99% of cases occurring in low-middle income countries (Puzari *et al.*, 2018). As per the Global Antimicrobial Resistance & Use Surveillance System (GLASS) Report in 2022, among 3,723 bacteriologically confirmed *Shigella* infections reported in 34 countries, territories, and areas, 3,109 showed resistance to at least one or more antimicrobial agents (World Health Organization, 2022). *S. flexneri* and *S. sonnei* were found to retain the status of the ‘most prevalent serogroup’ for a period of 5 years between 2017 and 2021, as pointed out in the annual report of Antimicrobial Resistance Research & Surveillance Network of India in 2021 (AMR Surveillance Network, 2021). A consistent decrease in the susceptibility of *S. flexneri* to an array of established antimicrobials, ranging from first-line drugs, such as ampicillin, to advanced 3^rd^ generation cephalosporins, including cefixime, was recorded between 2017 and 2021. The presence of AMR genes, including dhfrA, sulII, ampC, qnrS, qnrB, bla_OXA_, bla_CTX-M_, and bla_TEM_, identified using PCR characterization in 37 *Shigella* isolates cumulated from varied zones of India highlights the need to scale up AMR surveillance (AMR Surveillance Network, 2021).

## Materials and Methods

### Sampling and bacterial isolation

A liquid sludge sample was collected from the main drainage system (receiving untreated hospital effluents, domestic waste water, and agricultural runoff) in a rural area of Purulia district, West Bengal, India. The sample was collected in sterile containers maintaining strict aseptic protocols, transferred to the laboratory (within 10‍ ‍h of collection), and stored at –20°C. The collected sample was subjected to membrane filtration to remove soil particles and other granular entities, following which it was serially diluted 10-fold (10^–1^–10^–6^). One milliliter of the filtered stock solution was removed and diluted with 9‍ ‍mL of distilled water, and this step was subsequently repeated until a 10^–6^ gradient was obtained. One hundred microliters of the resulting dilutions were plated on nutrient agar (NA) and incubated at 37°C for up to 48 h. Plates with a countable number of discrete isolated colonies were closely observed to identify morphologically distinct colonies, which were picked up and freshly plated.

We then performed biochemical tests that are known to produce confirmatory results for *Shigella* as a primary step to identify the pathogen of choice from the pure bacterial cultures obtained. A bacterial strain exhibiting results exemplified by *Shigella* was subjected to genomic DNA extraction according to the method of Chen and Kuo (1993). Isolated microbial DNA was selectively amplified for the 16SrRNA gene using the two universal primers 27f [5′-AGAGTTTGATCCTGGCTCAG-3′] and 1492r [5′-TACGGTTACCTTGTTACGACTT-3′]. Amplified PCR products was then sequenced to further confirm that the bacterial isolate was *Shigella*.

### Phenotypic assays for antimicrobial susceptibility testing

The Kirby-Bauer disc diffusion assay was performed according to CLSI standards (33^rd^ edition) in order to evaluate the susceptibility of the isolated strain against different antibiotic classes (CLSI, 2023). The bacterial isolate was cultured overnight in Mueller-Hinton (MH) broth. Eighteen hours later, 200‍ ‍μL of the enriched culture was transferred to a nephelometric flask containing 20‍ ‍mL of MH broth and incubated at 37°C with shaking. The optical density (O.D.) at 600‍ ‍nm of the nephelometric flask was measured at intervals of 1 h. Upon reaching an O.D. value of 0.5 units, fresh MHA plates were inoculated with 200‍ ‍μL of the bacterial suspension using spread plate techniques, and antibiotic discs were immediately placed, one on each quadrant. After an incubation at 37°C for 24 h, measurements of zone diameters were recorded with supporting images.

### WGS ana­lysis

The microbe was freshly grown in an overnight culture for the purpose of genomic DNA extraction, which was conducted according to the protocol of Chen and Kuo (1993). Extracted genomic DNA was dispatched to Molsys Pvt. for WGS. Sequence-generated reads were quality checked and filtered using FASTQC software, and trimmed reads were assembled using the unicycler program in the KBase data science platform, guided by the reference genome NC-004337 (Leggett *et al.*, 2013; Wick *et al.*, 2017). The complete genome sequence data of isolated *S. flexneri* (SFMMGSG_23) is accessible using Accession CP123365 at the National Center for Biotechnology Information (NCBI). The draft genome was annotated using the Prokka tool V.1.1.0 and Prokaryotic Genome Annotation Pipeline (PGAP) of NCBI (Seemann, 2014; Tatusova *et al.*, 2016). Probable antibiotic resistance genes embedded within the isolated strain were detected by browsing the Comprehensive Antibiotic Resistance Database (CARD) (https://card.mcmaster.ca/; accessed on 27^th^ April 2023 at 12:25 P.M. IST) using the Resistance Gene Identifier (RGI) tool (Alcock *et al.*, 2020). A high quality interactive map of the complete circular genome was generated using the Proksee-Genome Annotation system (Grant *et al.*, 2023). Open reading frames spanning different lengths of the genome along with the coding sequences were predicted using the ORF feature of the Proksee server (Grant *et al.*, 2023). The occurrence of lateral gene transfer events and the presence of transposable elements within the bacterial genome were inferred using the web-based applications Alien-Hunter and mobileOG database (mobileOG-db [vt.edu]; accessed on 27^th^ April 2023 at 10:18 A.M. IST), respectively (Vernikos and Parkhill, 2006; Brown *et al.*, 2022). All data were generated by providing the whole genome sequence of SFMMGSG_23 in the GenBank format and keeping the parameters set at default values. To identify the number and types of secondary metabolites encoding biosynthetic gene clusters (BGCs), the draft genome of the isolated pathogen was analyzed using antiSMASH version 7.0 (Blin *et al.*, 2023).

### Comparative pangenomics

Fifteen different variants of *S. flexneri*, reportedly isolated from seven different south-east Asian countries (China, Bangladesh, Singapore, Hong Kong, India, Taiwan, and South Korea) were selected by mining the Assembly database of NCBI (Kitts *et al.*, 2016) (Table 1).

The complete genome sequences of the selected strains were retrieved from the Genbank database. Sixteen strains (1 isolated; 15 selected) were subjected to a pangenome analysis based on clustering of orthologous protein sequences, using a perl based software called Bacterial Pan Genome Analysis (BPGA) (Chaudhari *et al.*, 2016). Protein sequences were clustered using the USEARCH algorithm, adjusting for a sequence identity cut-off of 50% (default parameter). BPGA allows for pangenome construction by the sequential addition of genomes in 30 different combinations to avoid any bias and calculates the core and pangenome size by considering the mean values for each (Chaudhari *et al.*, 2016). A reference genome-free phylogenetic tree was generated based on 20 randomly selected clusters of shared genes between these 16 strains using the same BPGA tool.

### Pathway enrichment ana­lysis

The set of genes comprising the core genome was mapped to canonical biological pathways using the KEGG Automated Annotation Server (KAAS) with a default threshold bit-score value of 60, the bi-directional best hit method, and the BLAST program (Moriya *et al.*, 2007). KAAS assigns individual protein sequences with KEGG orthology (KO) identifiers, and allows pathway reconstruction by establishing a direct link between a cluster of orthologous genes (COG) and an object in the KEGG pathways and BRITE functional hierarchy.

## Results and Discussion

### Antibiotic susceptibility profile

The present study successfully isolated and purified a strain of *S. flexneri* from the liquid sludge sample collected. The isolated strain was examined for its susceptibility to antibiotics belonging to diverse classes and generations. The *in vitro* susceptibility profile of the selected strain revealed resistance to all 11 antibiotics used, thereby confirming the isolate as MDR (Table 2).

### Genomic features of isolated *S. flexneri*

WGS using the Illumina HiSeq platform generated 12,000‍ ‍Mb of raw sequence reads, which were quality checked and clean reads were assembled into a circular genome with a length of 4,607,202 base pairs. The genome (SFMMGSG_23) has been deposited to NCBI under the Accession CP123365. PGAP of NCBI predicted 4,640 genes, 3,845 of which were protein-coding genes, while 127 were genes coding for different RNAs and the remaining 668 were regarded as pseudogenes that either had ambiguous residues or may result in truncated peptides. Proksee was then used to analyze the draft genome, which employs different tools to evaluate genomic features with the primary DNA sequence as the starting material and allows for the visualization of sequence features on interactive graphical maps (Supplement Fig. S1).

The identification of coding components in the genome via Prokka estimated 4,834 coding genes, 2,830 of which were assigned to distinct COG categories, while 1,105 were found to code for hypothetical proteins. A functional enrichment ana­lysis of the 2,830 coding genes with COG definitions revealed genes belonging to 23 distinct COG cate­gories, suggesting their involvement in the manipulation of differ­ent facets of pathogen survival and evolution (Supplement Fig. S2).

**Fig. 1.**
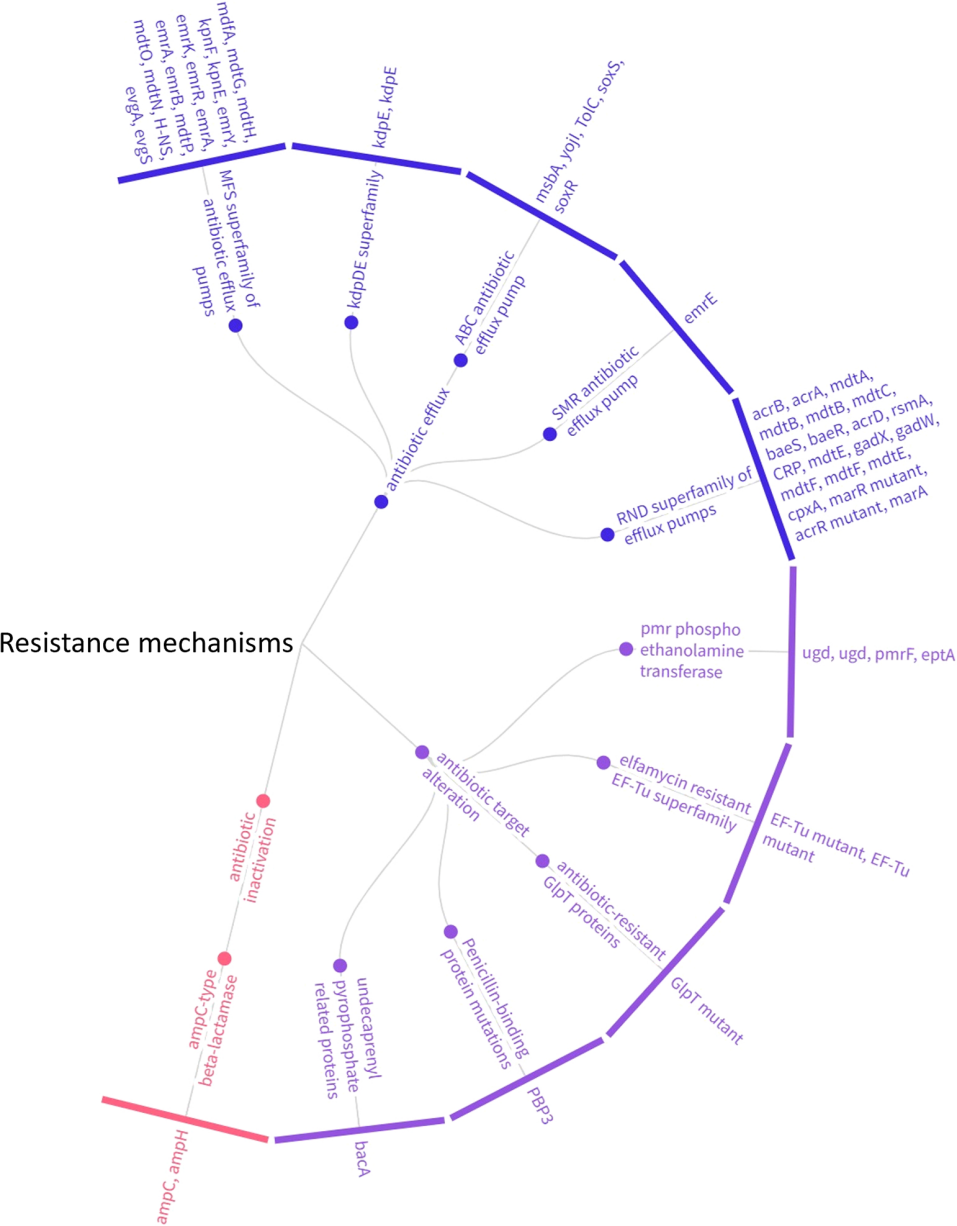
A hierarchy chart summarizing AMR genes across different categories based on their mechanism of action.

Fifty-seven AMR genes belonging to 11 unique gene families were identified in a search against the CARD database using the RGI tool, with more than 50% of the genes coding for antibiotic efflux proteins and 15% coding for proteins involved in target modification (Fig. 1).

Annotation of the draft genome using mobileOG-db revealed 826 sequences, mediating the different essential life cycle functions of MGEs (Supplement Fig. S3). Overall, 484 of these elements were efficient for integration/excision from one genetic locus to another, 137 were associated with bacteriophage-related life cycle processes, 118 were able to initiate the replication, recombination, and repair of nucleic acids, 47 were capable of interbacterial transfer, and 40 were implicated in the stability and defense of these elements (Supplement Fig. S3).

**Fig. 2.**
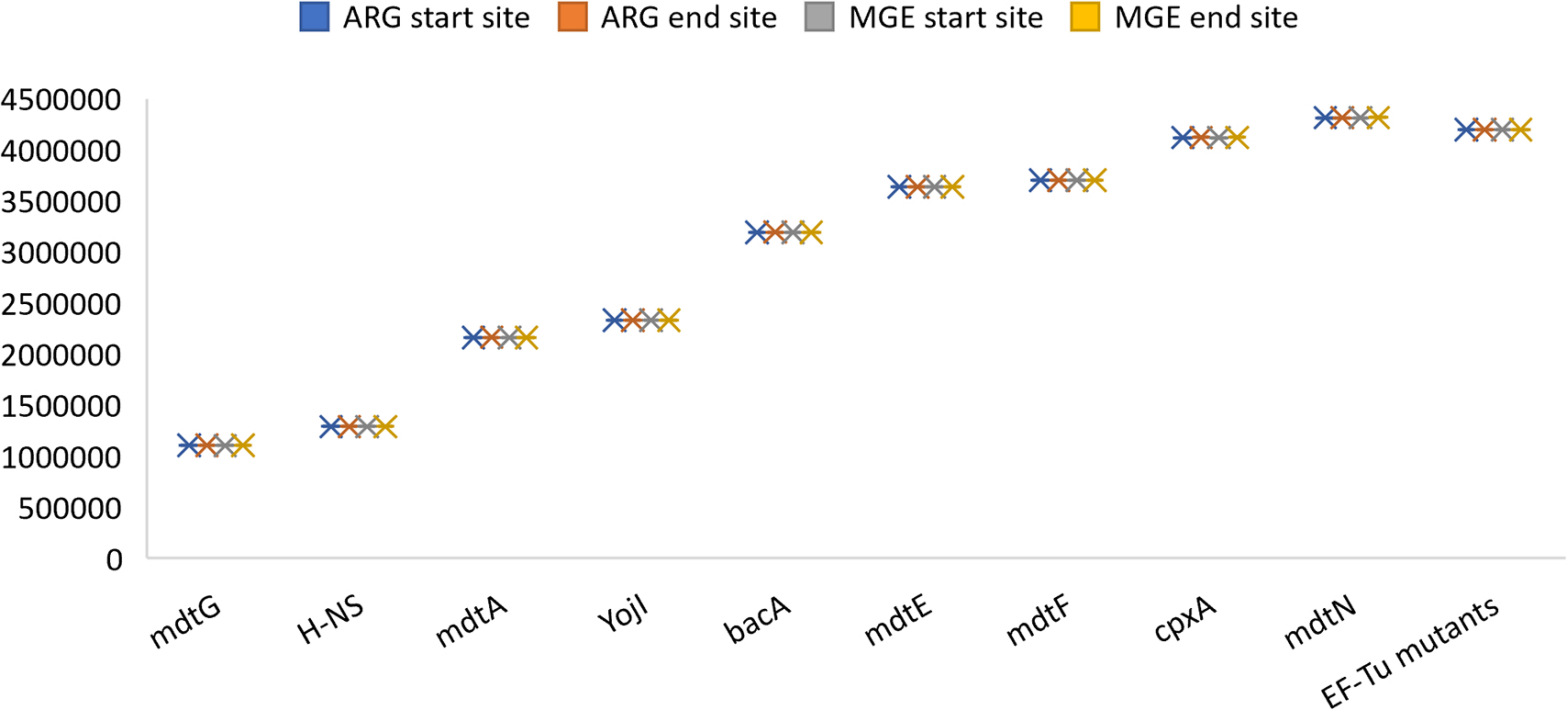
A box and whisker chart showing positional nearness between identified ARGs and MGEs.

To further correlate the occurrence of ARGs with the presence of MGEs, we investigated the positional nearness of the ARGs detected with the predicted MGEs, and found that 10 ARGs were in close proximity to the predicted MGEs, strongly suggesting the plausible transfer of resistance genes via MGEs (Fig. 2).

Five of the 10 closely related MGEs were associated with integration/excision, two were involved in the recombination of nucleic acids, two exhibited a resemblance to phage-related genes, and one showed a connection to conjugation-mediated transfer processes, thereby tracing the different pathways for the gradual inclusion of resistance genes within bacterial genomes.

**Fig. 3.**
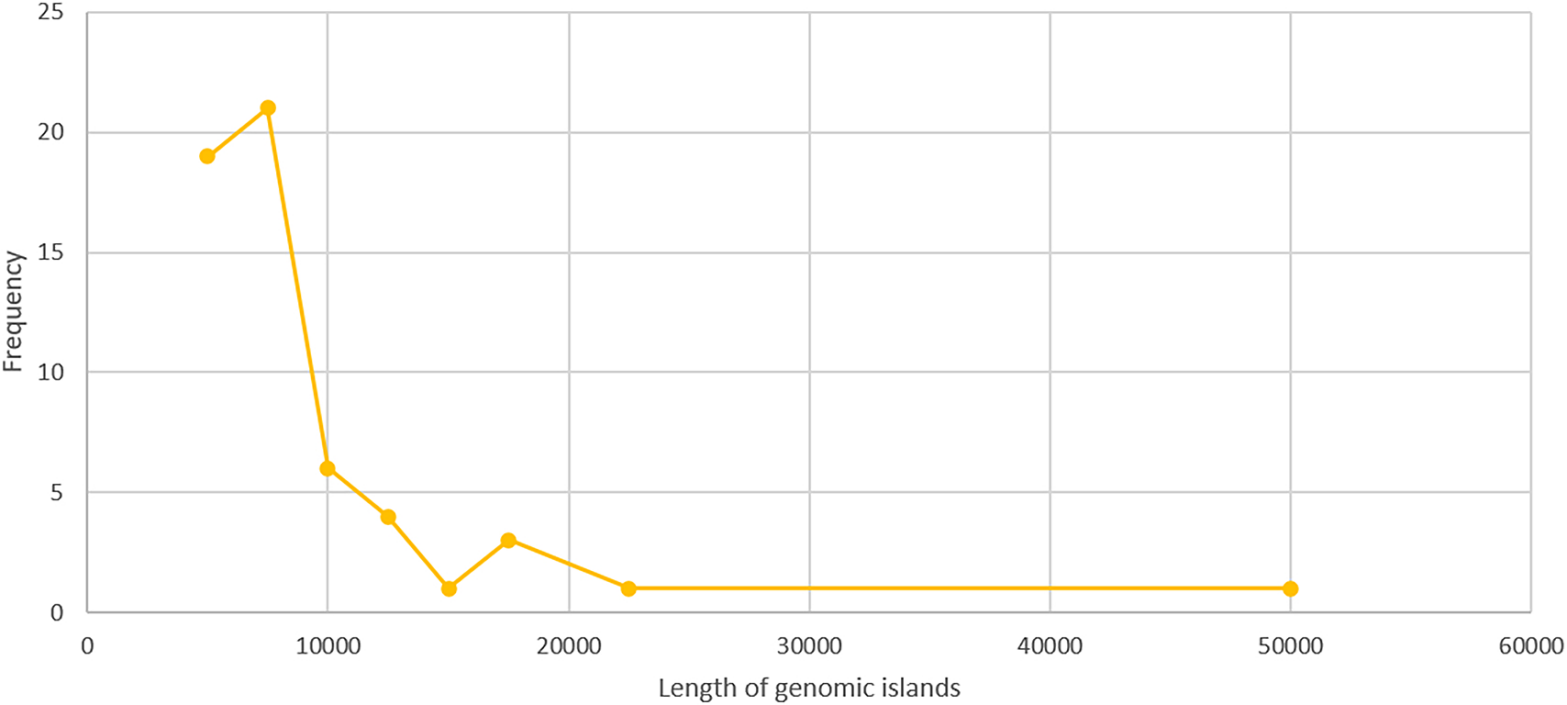
A scatter chart mapping the relationship between the length of horizontally acquired genomic regions and their relative frequency

Scanning MGEs from whole-genome sequences is important for inferring the extent of horizontal gene transfer events that are critical drivers of genomic diversity and pathogen evolution. AlienHunter was utilized to delineate the occurrence of horizontally transferred genomic regions within the isolated pathogen. This tool selects atypical genomic regions based on different compositional metrices (such as the GC content, codon/amino acid bias, and dinucleotide frequency) by accounting for both the distribution and possible combination of variable order motifs ranging from 1–8 base pairs (Vernikos and Parkhill, 2006). We identified a large repertoire (56) of horizontally transferred genomic candidates from the isolated bacteria, which accounted for approximately 11% of its genomic content. An evaluation of the relative frequency of these alien regions in terms of their sequence length revealed an inverse correlation between the two, indicating the relative ease of stabilization and vertical transmission of smaller inserts (Fig. 3).

**Fig. 4.**
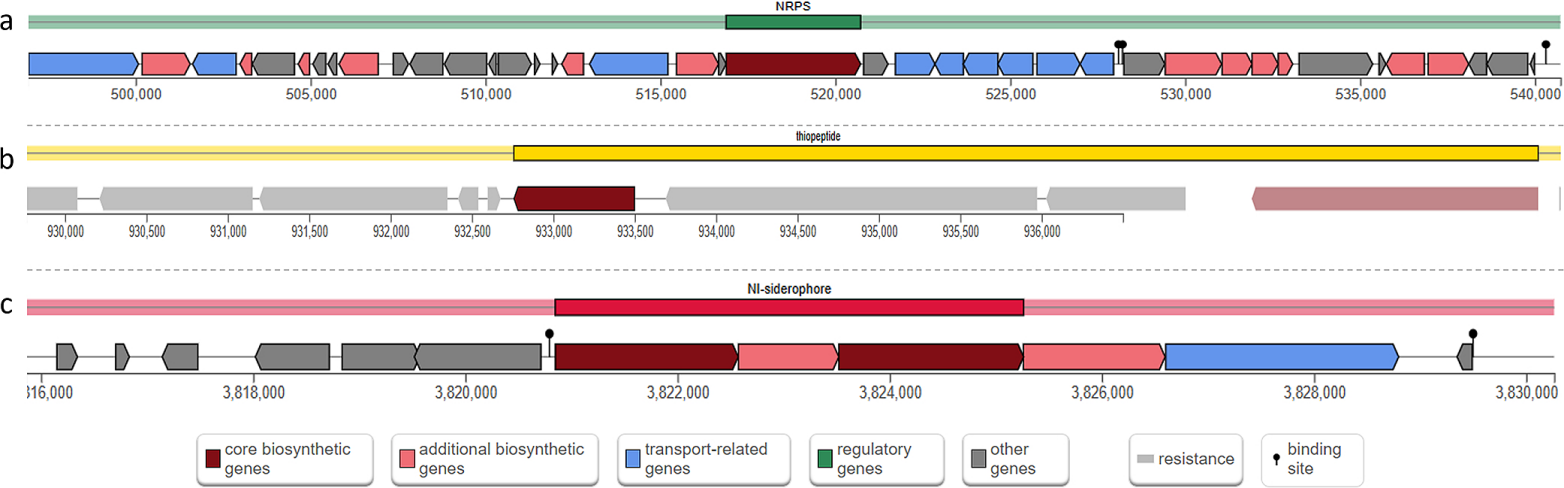
Schematic representation of the distribution of biosynthetic gene clusters across the assembled genome of SFMMGSG_23; (a) non-ribosomal peptide synthetase (NRPS), similar to the enterobactin biosynthetic gene cluster from *Escherichia coli* str. K-12 substr. MG1655; (b) thiopeptide, similar to the O-antigen biosynthetic gene cluster from *Pseudomonas aeruginosa*; (c) NRPS-independent (NI), IucA/IucC-like siderophores, similar to the aerobactin biosynthetic gene cluster from *Pantoea ananatis*. (The colors of BGCs and coding sequences are autogenerated by the server used)

Microbial secondary metabolites encoded by BGCs serve as a rich source of natural chemicals and are considered to play roles in host-pathogen interactions. To elucidate these possibilities, the isolated genome was examined for the presence of BGCs using the stand-alone tool AntiSMASH. The genome was found to house 3 BGCs, which were involved in the production of diverse iron-chelating agents and thiopeptides (Fig. 4).

Two of the identified BGCs code for enzymes (including EntF, IucA, and IucC) involved in the synthesis and internalization of siderophores of two chemical types, *viz.* hydroxamic acids (aerobactin) and catechols (enterobactin). High-affinity iron-sequestering systems are capable of solubilizing and extracting ferric iron from insoluble complexes and host iron-binding proteins, and thereby endow the pathogen with the skill to survive iron-deficient environments within the host (Sheldon *et al.*, 2016). Besides iron acquisition, siderophores also play important roles in tolerance to oxidative stress, the detoxification of reactive oxygen species (ROS), and the promotion of virulence. In a‍ ‍recent study on aerobactin activity in *Yersinia pseudotuberculosis* YPIII, infection in a mouse model with mutant aerobactin biosynthesis genes resulted in a significant reduction in lethality rates and smaller bacterial loads than in mice infected with wild-type strains, which confirmed the contribution of siderophores in microbial colonization and pathogenicity (Li *et al.*, 2021). The genome was also found to host putative thiazolyl-like biosynthetic genes, many of which have been regarded as signaling molecules, affecting bacterial phenotypes in a manner relevant to both humans and the environment. In a recent study that exami­ned the non-antibiosis potential of thiopeptides, thiocillins secreted by *Bacillus cereus* induced the expression of biofilm genes in *Bacillus subtilis* (Bleich *et al.*, 2015). A combination of co-cultures, fluorescent reporter assays, and imaging mass spectrometry revealed that this biofilm induction potential may be a regular feature of structurally diverse thiopeptides produced by different bacterial species. The identification of thiopeptide synthesis genes in *Shigella* will provide an opportunity to investigate whether and how novel thiopeptides act as chemical cues that have an impact on the distribution and phenotypes of medically significant pathogens.

### Pangenome coverage and dynamicity in the genome composition

**Fig. 5.**
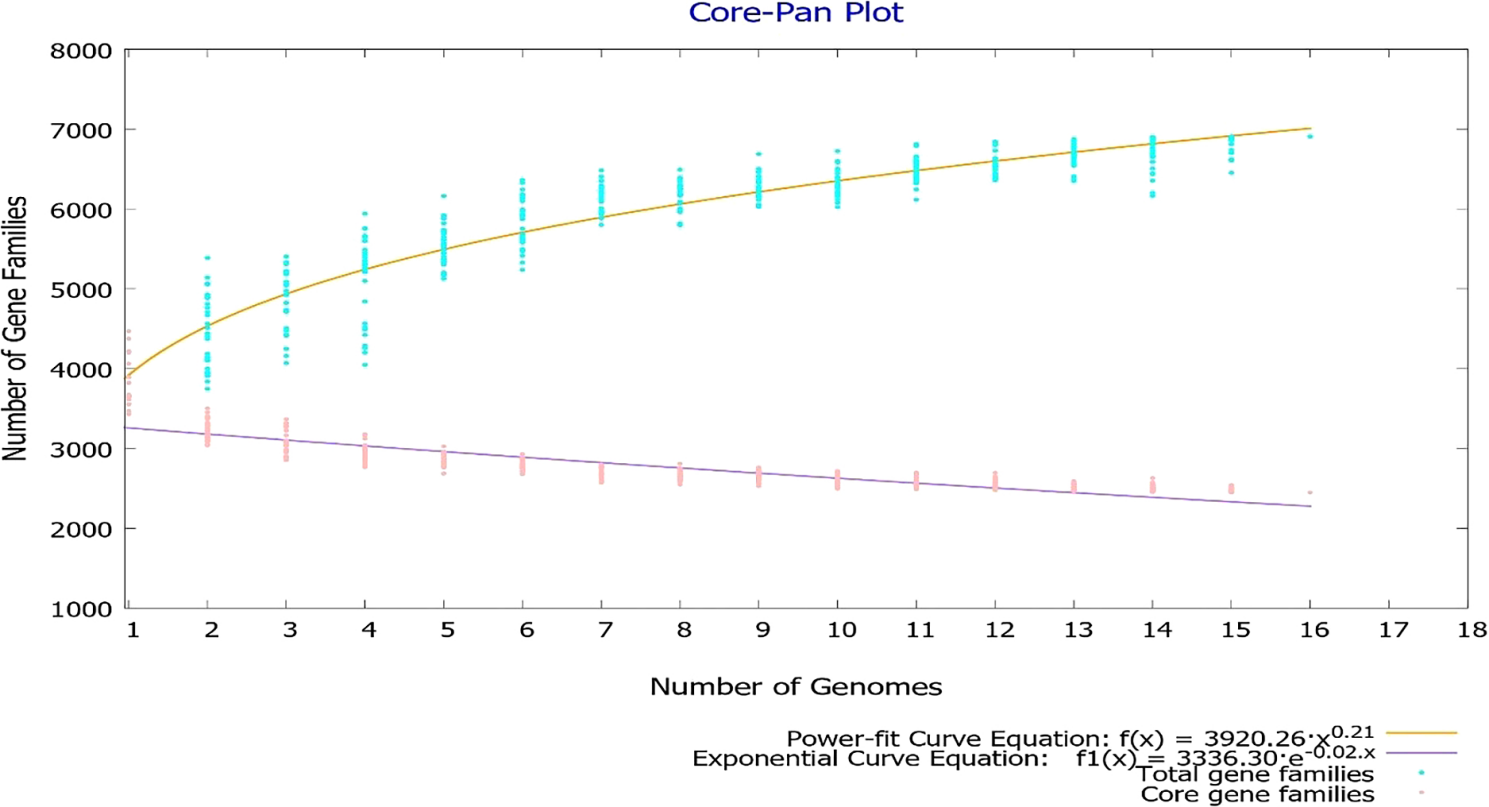
Graph portraying the effect of the dataset size on pan/core genome dimensions

To comprehend the adaptive capacity and genomic variability of *S. flexneri*, 15 *S. flexneri* strains with the highest assembly level, collected across seven sites in south-east Asia, were analyzed using BPGA software. During the initial stage of input preparation, BPGA annotated 69,177 sequences originating from the 15 bacterial strains, which were subsequently clustered using the USEARCH algorithm. Clustering revealed a pangenome size of 6,907 gene families, 2,448 (~35%) of which contained at least one member from each genome, thereby comprising the core genome of *S. flexneri*. The accessory genome shared by at least 2 strains harbored 2,759 (~40%) gene families, whereas the unique genome included 1,700 gene families, accounting for approximately 25% of the entire gene pool. The large percentage of unique and accessory genes (~64%) reflects the intraspecies heterogeneity of *S. flexneri* and explains the fast escape of infection-induced immunity against related *S. flexneri* serotypes. As shown in the pangenome curve, the total repertoire of gene families steadily increased with the addition of each new genome, which is consistent with the value of ‘b’ (b=0.209597) extrapolated using the power law equation, n=aN^b^, (n: pangenome size; N: number of genomes, a and b: fitting parameters) where b>0 indicates an open pangenome (Fig. 5).

Contrasting results were observed for core genes, with gradual decreases in their numbers as the genome count increased. Although we observed a decline in the rate of increases in pangenome dimensions, neither the pangenome nor core genome sizes attained a stationary phase, implying more room for the addition of new genes with further genome inclusions. The dynamic genome composition, as evidenced by fluctuating pan/core genome sizes, indicates the fluid nature of core genes, with their inclusion/exclusion being dependent on the complex interplay between the genetic background and environment.

**Fig. 6.**
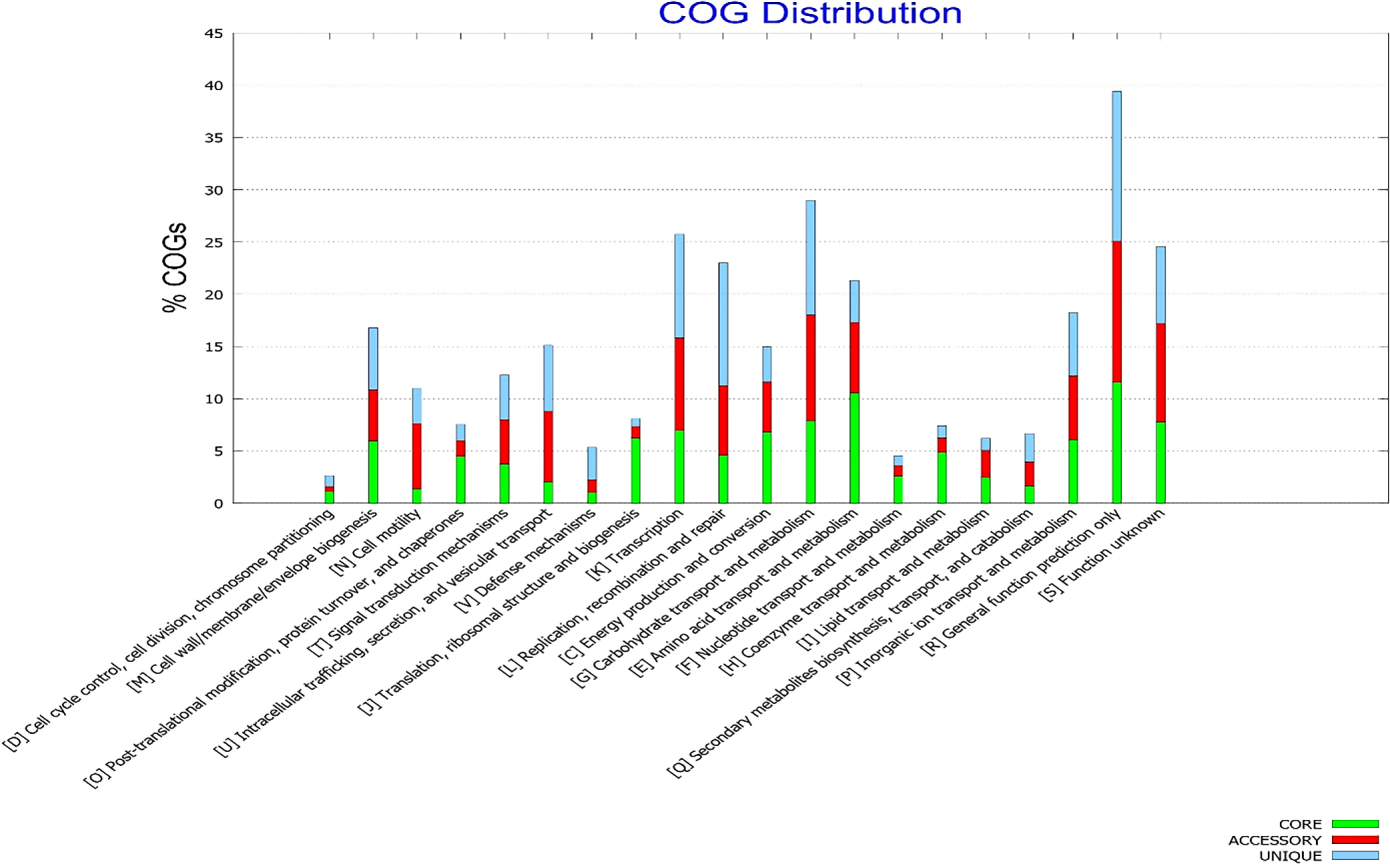
Functional breakdown of core, accessory, and unique genes into different COG categories

To interpret the relationship between gene frequency and their associated functions, all genes were annotated using the Pangenome Functional Analysis Application of the BPGA program. The results obtained clearly showed a biased distribution of core genes involved in metabolic processes, with more than 5% of core genes under each COG category, *viz.* COG E (amino acid transport and metabolism), COG G (carbohydrate transport and metabolism), COG C (energy production and conversion), and COG H (coenzyme transport and metabolism) (Fig. 6).

**Fig. 7.**
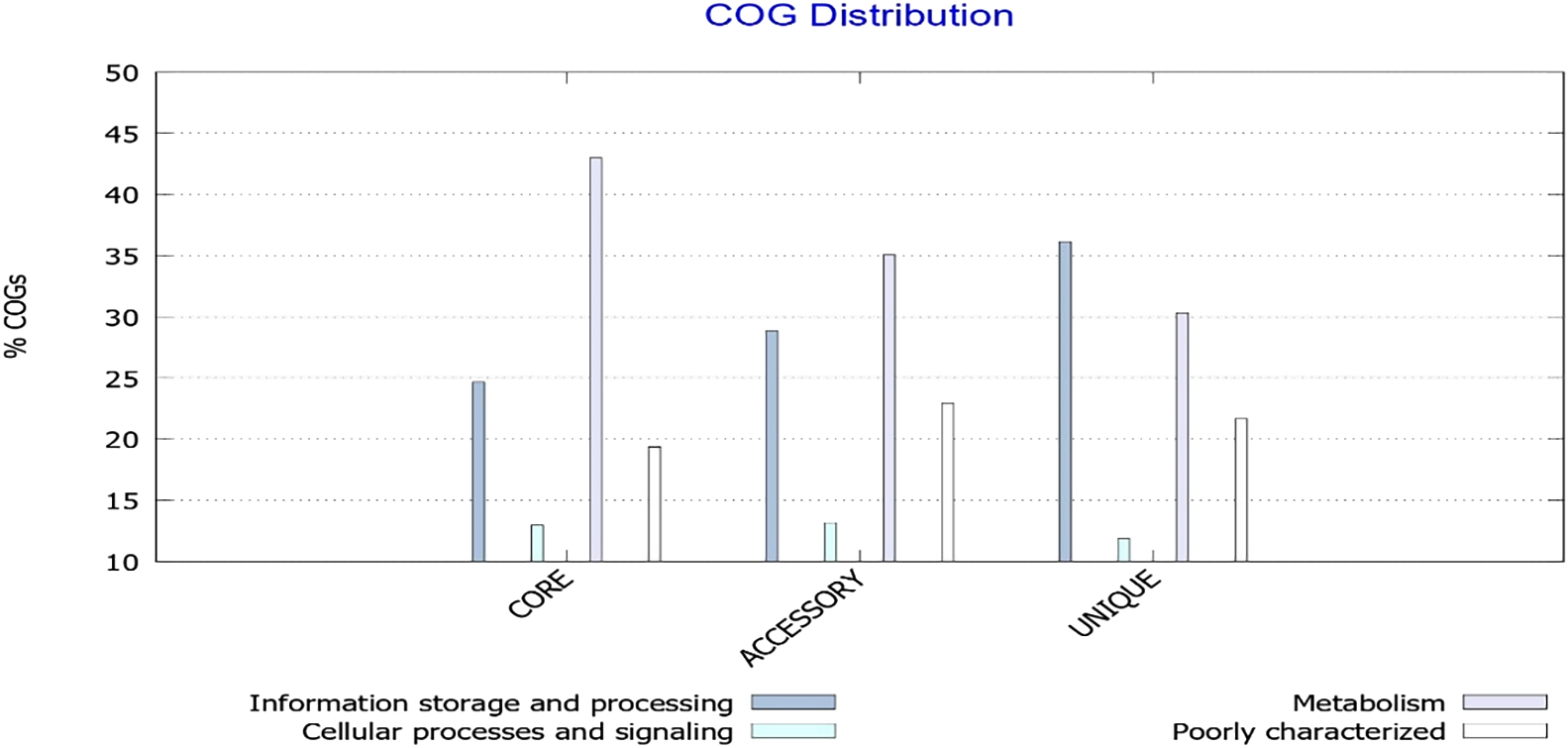
Bar plots showing the average distribution of COG classes in core, accessory, and unique genomes of 16 annotated *Shigella flexneri* strains

The opposite results were observed for the COG profiles of the unique genome, with an appreciable number of genes coupled to information storage and processing, demon­strating an involvement in intracellular trafficking, secretion and vesicular transport, the synthesis and distribution of secondary metabolites, and defense mechanisms (Fig. 7). The COG ana­lysis of our pangenome collection indicated an overall enrichment of universal genes in cellular processes that are critical for pathogen survival under any conditions, while strain-specific unique and accessory genes had more niche roles, such as the biogenesis of cell wall components and the synthesis and secretion of virulence factors, which accounted for the majority of the observed serotype diversity.

**Fig. 8.**
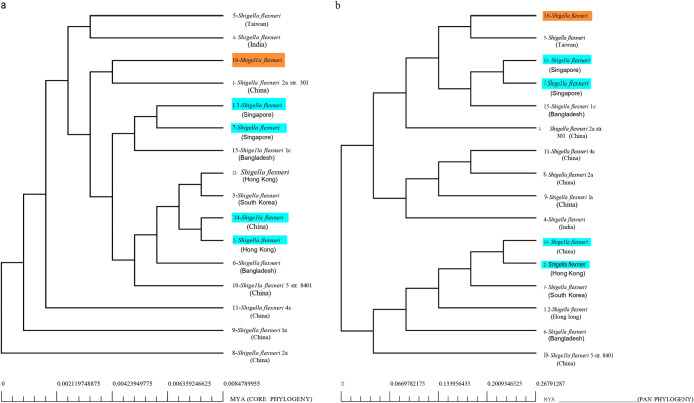
Evolutionary hierarchy of 16 *Shigella flexneri* strains based on (a) core genes; (b) the pan matrix. Recurrent clustering patterns are highlighted in blue. The isolated strain is highlighted in orange

A comprehensive phylogenetic inference based on concatenated core gene alignments and the presence/absence pattern of pan genes was performed to investigate the evolutionary relationship between the 16 *S. flexneri* strains. An automated alignment of 20 random clusters of core genes allowed for the construction of a neighbor-joining phylogenetic tree that showed no definitive pattern of association between the phylogenetic distance and the country/time of origin of strains (Fig. 8a). The pan matrix-based phylogenetic tree had deviations from the previous tree, but did not delineate any specific correlation (Fig. 8b). Two pairs of strains (13,7 and 14,2) were predicted to be phylogenetically close by both approaches, with strains 7 and 13 being collected from Singapore in 2022 and strains 14 and 2 being collected from China and Hong Kong in 2020 and 2018, respectively. The MDR *S. flexneri* isolated and analyzed in the present study belonged to the same branch as strain 1 (*S. flexneri* 2a strain 301) from China as per core genome alignments, whereas it was the closest to strain 5 (*S. flexneri* strain: 3160_NCHU22) from Taiwan based on the binary pan genome matrix. This result suggests that an equivalent selection pressure, exerted by similar environmental parameters, may have resulted in the evolution of common phenotypes that were further affected by horizontal gene transfer and serotype switching (Blount *et al.*, 2018).

### KEGG enrichment of core genes

**Fig. 9.**
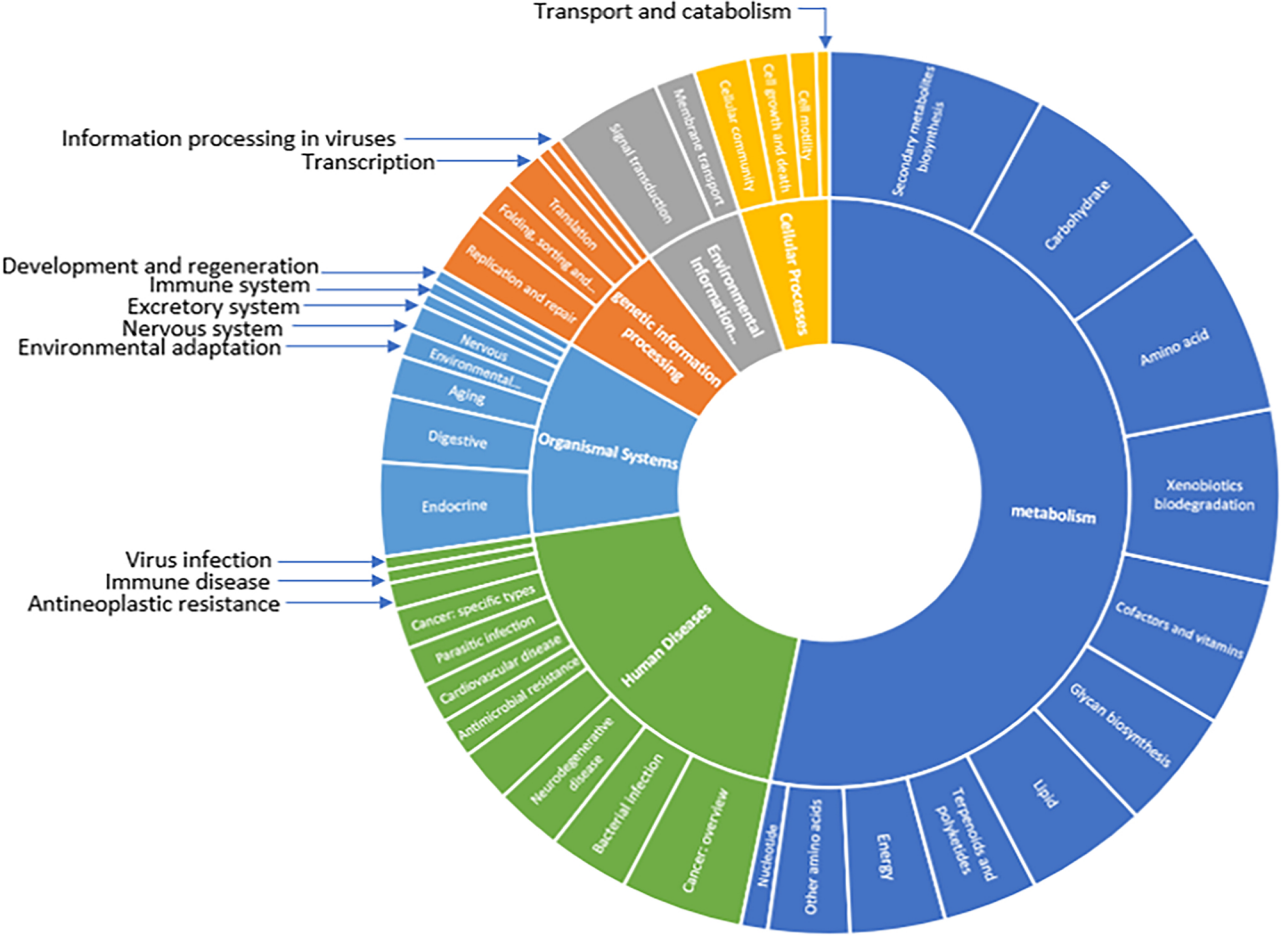
KEGG pathways affected by core genes

A pathway ana­lysis of the core genomic factors (2,448 core genes) obtained revealed 205 affected pathways, with the majority being metabolic processes, followed by 40 human disease pathways, 22 pathways related to organismal systems, 13 associated with genetic information processing, 11 for environmental information processing, and 10 linked to cellular processes (Fig. 9).

While the core genes were expected to be enriched for housekeeping functions fundamental to cellular maintenance, we focused on the presence of drug resistance pathways with significant gene enrichment that have been embodied in the core genome. KAAS pathway mapping of 2,448 core genes revealed 2 resistance pathways, containing 22 uniquely identifiable genes capable of directly or indirectly affecting AMR (Table 3). The dedicated resistance functions encoded by the core genomic elements included multidrug efflux pumps, outer membrane permeability proteins, and two-component systems regulating gene expression in response to antibiotic stress.

The resistance pathways identified from our dataset hosted 6 genes homologous to the two-component signal transduction systems (TCSs), *viz.* PhoQ, PhoP, PmrB, PmrA, CpxA, and CpxR (Supplement Fig. S4). TCSs typically consist of a sensor histidine kinase (HK) and a cytoplasmic response regulator (RR) that are capable of reprogramming gene expression, favoring microbial adaptation to different host milieus. Recent studies reported the‍ ‍involvement of two TCSs, PmrA/B and PhoP/Q, in conferring resistance to polymyxins and a range of cationic antimicrobial peptides through lipid A modifications. Polymyxins, which are known to disrupt bacterial outer membranes by inserting fatty acyl tails into lipid leaflets, initially exert their effects using positively charged L-α,γ-diaminobutyric acid residues to interact with the negatively charged phosphate groups of lipid A. The PmrA/B protein machinery present in the core genome of our analyzed strains was capable of sensing external signals (high concentrations of Fe^3+^ and Al^3+^ and low pH) and responded by inducing the transcription of the lipid A modification gene eptA (detected in our dataset), which introduced positively charged groups (phosphoethanolamine) on lipid A, thereby reducing its affinity for polymyxins (Bhagirath *et al.*, 2019; Huang *et al.*, 2020). Similarly, the PhoP/Q regulatory system also perceived the presence of polymyxin B/colistin in the periplasm and, in turn, activated the expression of the downstream gene PagP, coding for palmitoyltransferase (one of the resistance factors in our set of core genes), which enforced the palmitoylation of lipid A, resulting in resistance to polymyxins. Previous studies revealed the presence of a vast number of anonymous mutations in the PmrA/B and PhoP/Q systems, which make them constitutively active, fostering polymyxin resistance (Huang *et al.*, 2020). The CpxA/R genes identified by the KAAS annotation were also found to play important roles in conferring resistance to β-lactams in multiple clinically relevant enteropathogens. The cell wall imbalance caused by β-lactams binding to penicillin-binding proteins (PBP1a/2, PBP2, and FtsI genes detected in our dataset) induced the expression of genes of‍ ‍the Cpx regulon, *viz.* the DegP-protease/chaperone (identified in our dataset), PpiA-peptidyl-propyl-isomerase (present within the pool of core genes), and AcrD-efflux pump, which resulted in a response to envelope stress either by degrading misfolded proteins (DegP) or eliminating the drug (AcrD) (Masi *et al.*, 2020).

Although various mechanisms underlie the origin of resistance behavior in bacterial populations, the abundance of chromosomally encoded efflux pumps in Gram-negative pathogens is one of the fastest acting and most efficient resistance mechanisms (Du *et al.*, 2018). In our repertoire of core genomic elements mapped to different resistance pathways, a large number of genes were found to code for‍ ‍multidrug efflux pumps. The presence of genes coding for an outer membrane channel (TolC), inner membrane transporter (AcrB), and their periplasmic counterpart, a membrane fusion protein (AcrA), which function as a coordinated network and comprise the tripartite Resistance Nodulation Division (RND) complex, is of clinical significance (Supplement Fig. S5). An increase in the efflux activity of RND pumps has frequently been associated with the occurrence of MDR in Gram-negative pathogens (Piddock, 2006; Puzari and Chetia, 2017; Colclough *et al.*, 2020). Efflux by the AcrAB-TolC RND system in *Escherichia coli*, was found to confer resistance to fluoroquinolones, β-lactams, tetracycline, erythromycin, chloramphenicol, novobiocin, and linezolid (Du *et al.*, 2018). The substrate polyspecificity of RND pumps is generally attributed to the AcrB component, which comprises a transmembrane domain (coupling a proton gradient with drug efflux), a pore domain (a portal for drug entry), and a docking domain (for pairing with AcrA). Recent advances in our structural understanding of RND pumps revealed the existence of two binding pockets, *viz.* a proximal (access) binding pocket and a distal (deep) binding pocket lodged in the pore domain, with varying antibiotic affinities based on substrate mole­cular weights, amino acid residues in the binding site, and the dynamicity of the binding loops (Blair *et al.*, 2014). AcrB functions as a homotrimer, with each monomer undergoing a conformational change upon drug binding and gradually transiting through 3 states: loose (L), tight (T) and open (O), in a cooperative manner that moves the drug further towards the outer membrane and simultaneously prevents its seepage back into the periplasm (Murakami *et al.*, 2006). Drugs of a high mole­cular weight are initially introduced into the proximal pocket during the L state and are gradually moved into the distal pocket during the L-T transition, while the distal pocket serves as the direct binding site for common antibiotics of a low mole­cular weight (Murakami *et al.*, 2006; Kobylka *et al.*, 2020). The structural change in AcrB during drug binding induces an organizational reform in the AcrA protein, resulting in an optimal fit between the helical hairpin domain of AcrA and the open TolC protein, thereby managing the effective extrusion of drugs (Murakami *et al.*, 2006). The overexpression of RND efflux pumps may be affected by the complex interplay between transcription factors or as a result of a spontaneous mutation arising within the transporter genes themselves in response to environmental signals (Piddock, 2006; Blair *et al.*, 2014). Recent studies reported that the expression of efflux pumps was controlled by TCSs, as evidenced by the involvement of CpxAR in activating multidrug efflux pumps in several members of Enterobacteriaceae (Dbeibo *et al.*, 2018). The co-occurrence of genes encoding both TCSs and multidrug efflux pumps as an integral part of the core genome indicated the hidden layers of adaptive responses that promote AMR in *S. flexneri*. The ubiquity and evolutionary conservancy of RND pumps and two component systems across multiple bacterial genera and their lack of conspicuous human homologs also makes them lucrative targets for future drug discovery.

## Conclusion

Shigellosis is again becoming an international disease of concern with increases in contaminated water systems worldwide. We herein characterized an XDR *Shigella* isolate from rural wastewater, which had antibiotic resistance genes in its genome, indicating a selection pressure that pushed ARGs into the genome. A pangenome ana­lysis established that genome variability in the selected isolates was attributed to the presence and absence of resistance gene cassettes with homeotic genes exhibiting conserved sequences. We consider the sequence of this genome to serve as an important reference genome for future comparative genomics and drug discovery studies and will share the strain with any interested researchers in the field.

## Citation

Mukhopadhyay, S., Singh, M., Ghosh, M. M., Chakrabarti, S., and Ganguli, S. (2024) Comparative Genomics and Characterization of *Shigella flexneri* Isolated from Urban Wastewater. *Microbes Environ ***39**: ME23105.

https://doi.org/10.1264/jsme2.ME23105

## Supplementary Material

Supplementary Material

## Figures and Tables

**Table 1.** Selected Genomes of *Shigella flexneri* from Asian Countries for a Pangenome Analysis

Serial no	Assembly Id	Date of submission	Collection date	Region of isolation	Species name	Strain Name
1	ASM692v2	8/3/2011	1984	China	*Shigella flexneri 2a*	str. 301
2	ASM2235470v1	2/22/2022	2018	Hong Kong	*Shigella flexneri*	Strain: SWHIN_101
3	ASM719759v1	7/20/2019	2019	South Korea	*Shigella flexneri*	Strain: C32
4	ASM217469v2	3/26/2019	2015	India	*Shigella flexneri*	Strain: FC906
5	ASM2286984v1	4/11/2022	2000	Taiwan	*Shigella flexneri*	Strain: 3160_NCHU22
6	ASM417118v1	2/12/2019	1984	Bangladesh	*Shigella flexneri*	Strain: SFL1520
7	ASM2473228v1	8/22/2022	2022	Singapore	*Shigella flexneri*	Strain: E
8	ASM158017v1	3/3/2016	1998	China	*Shigella flexneri 2a*	Strain: 981
9	ASM157812v1	3/1/2016	2002	China	*Shigella flexneri 1a*	Strain: 0228
10	ASM1358v1	7/21/2006		China	*Shigella flexneri 5*	Strain: 8401
11	ASM157996v1	3/3/2016	2012	China	*Shigella flexneri 4c*	Strain: 1205
12	ASM2249435v1	3/7/2022	2018	Hong Kong	*Shigella flexneri*	Strain: STLIN_17
13	ASM2473230v1	8/22/2022	4/13/2022	Singapore	*Shigella flexneri*	Strain: D
14	ASM1979357v1	8/29/2021	10/4/2020	China	*Shigella flexneri*	Strain: WW1
15	ASM244299v2	8/20/2019	1988	Bangladesh	*Shigella flexneri 1c*	Strain: Y394

**Table 2.** Antibiotic susceptibility patterns of isolated bacteria.

Antimicrobial agent	Disk content (μg disc^–1^)	Observed zone diameter±SE (mm)	Interpretive categories and zone diameter breakpoints based on CLSI standards (nearest mm)			Susceptibility
			S^1^	I^2^	R^3^	
Ampicillin	10	6.5±0.5	≥17	14–16	≤13	R
Tetracycline	30	6.5±0.5	≥15	12–14	≤11	R
Norfloxacin	10	6±0.6	≥17	13–16	≤12	R
Amoxycillin	30	7±0.5	≥18	14–17	≤13	R
Gentamicin	10	6±0.8	≥18	15–17	≤14	R
Amikacin	30	6.5±0.65	≥20	17–19	≤16	R
Cefoxitin	30	6.5±1.20	≥18	15–17	≤14	R
Imipenem	10	7.5±1.30	≥23	20–22	≤19	R
Cefuroxime	30	7.5±0.8	≥18	15–17	≤14	R
Trimeth-sulfa	25	6.5±0.5	≥16	11–15	≤10	R
Ciprofloxacin	5	6.5±1.20	≥31	21–30	≤20	R

^1^ S: Susceptible ^2^ I: Intermediate ^3^ R: Resistant

**Table 3.** Ensemble of genes associated with antibiotic resilient pathways (identified by assigning KO numbers to query genes, followed by gene mapping to biological pathways using the KAAS server).

pathway	gene	product
Beta-lactam resistance	AmpG	MFS transporter, PAT family, beta-lactamase induction signal transducer
	Opp	oligopeptide transport system substrate-binding protein
	OMP	outer membrane protein
	RND	multidrug efflux pump
	MFP	membrane fusion protein, multidrug efflux system
	PBP1a/2	penicillin-binding protein 1A
	PBP2	penicillin-binding protein 2
	FtsI	penicillin-binding protein 3
	MexA/AcrA	multidrug efflux system
	MexB/AcrB	multidrug efflux pump
Cationic antimicrobial peptide (CAMP) resistance	PhoQ	two-component system, OmpR family, sensor histidine kinase PhoQ
	PhoP	two-component system, OmpR family, response regulator PhoP
	PmrB	two-component system, OmpR family, sensor histidine kinase BasS
	PmrA	two-component system, OmpR family, response regulator BasR
	SapB	cationic peptide transport system permease protein
	SapC	cationic peptide transport system permease protein
	SapD	cationic peptide transport system ATP-binding protein
	SapF	cationic peptide transport system ATP-binding protein
	CpxA	two-component system, OmpR family, sensor histidine kinase CpxA
	CpxR	two-component system, OmpR family, response regulator CpxR
	AcrA	membrane fusion protein, multidrug efflux system
	AcrB	multidrug efflux pump
