# Supplementary material for: Comparative Genomics and Characterization of Shigella flexneri Isolated from Urban Wastewater
Source: Microbes Environ. 2024 Jun 5;39(2):ME23105. doi: 10.1264/jsme2.ME23105 (PMC11220449; doi:10.1264/jsme2.ME23105)
Supplement: Supplementary file 1 — Supplementary Material [file 39_23105_s1.pdf]

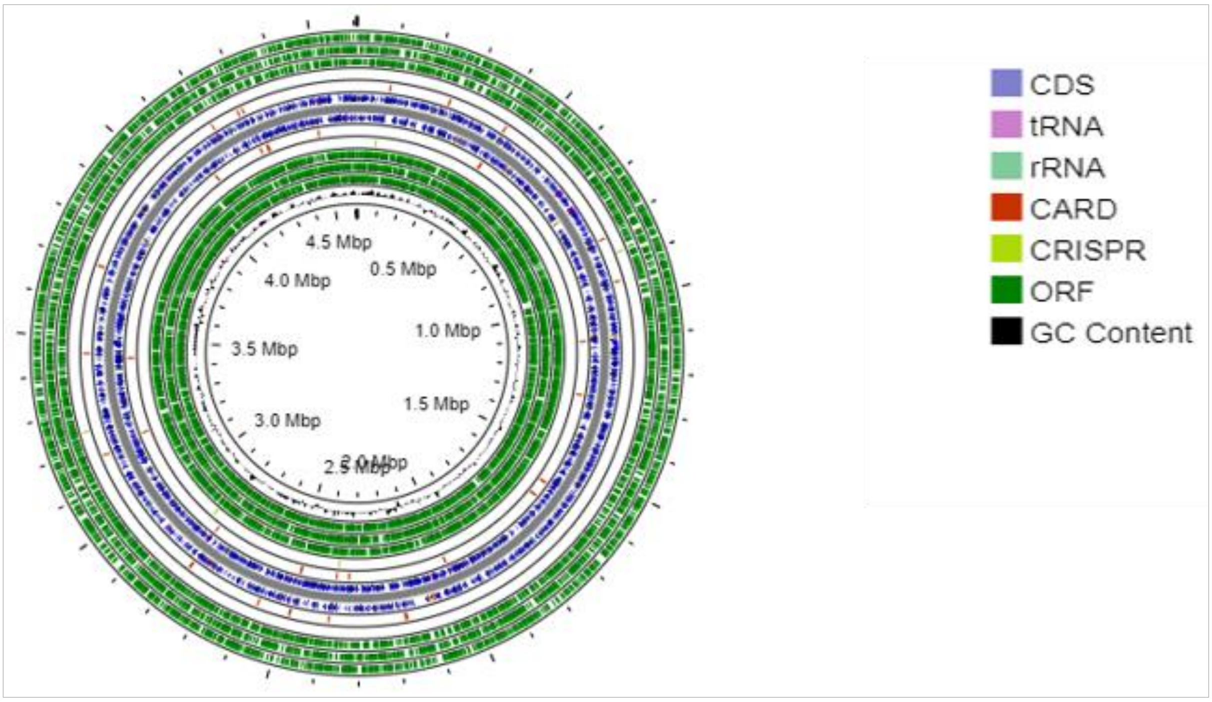

Supplement Fig. S1. Circular chromosome map of *Shigella flexneri* strain SFMMGSG_23, highlighting the number and spread of general genome features. A total of 9167 open reading frames (ORFs) are pointed out, with 4834 coding sequences, 57 resistance genes and 5 CRISPR arrays.

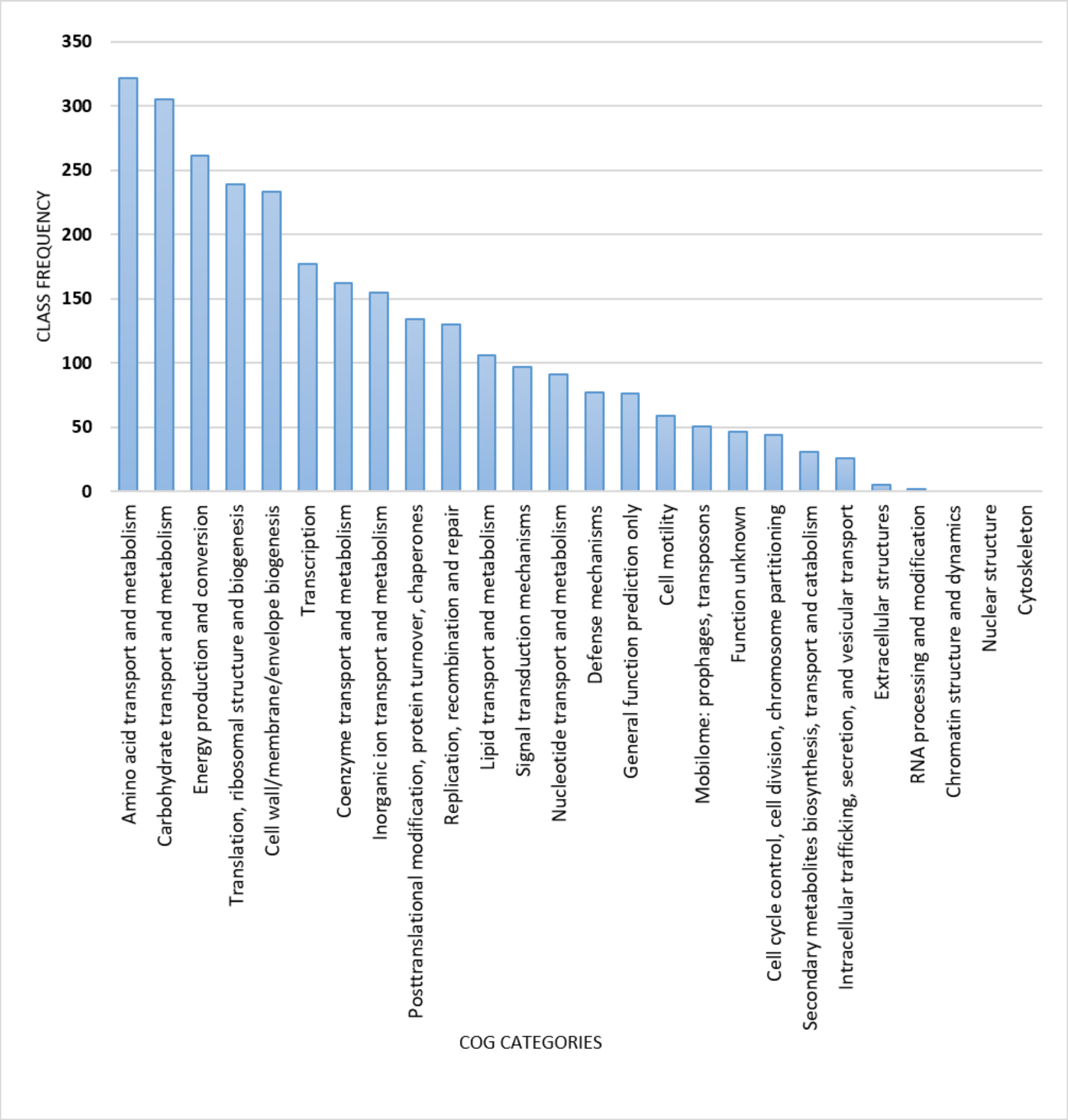

Supplement Fig. S2. Distribution of coding genes into different functional COG categories.

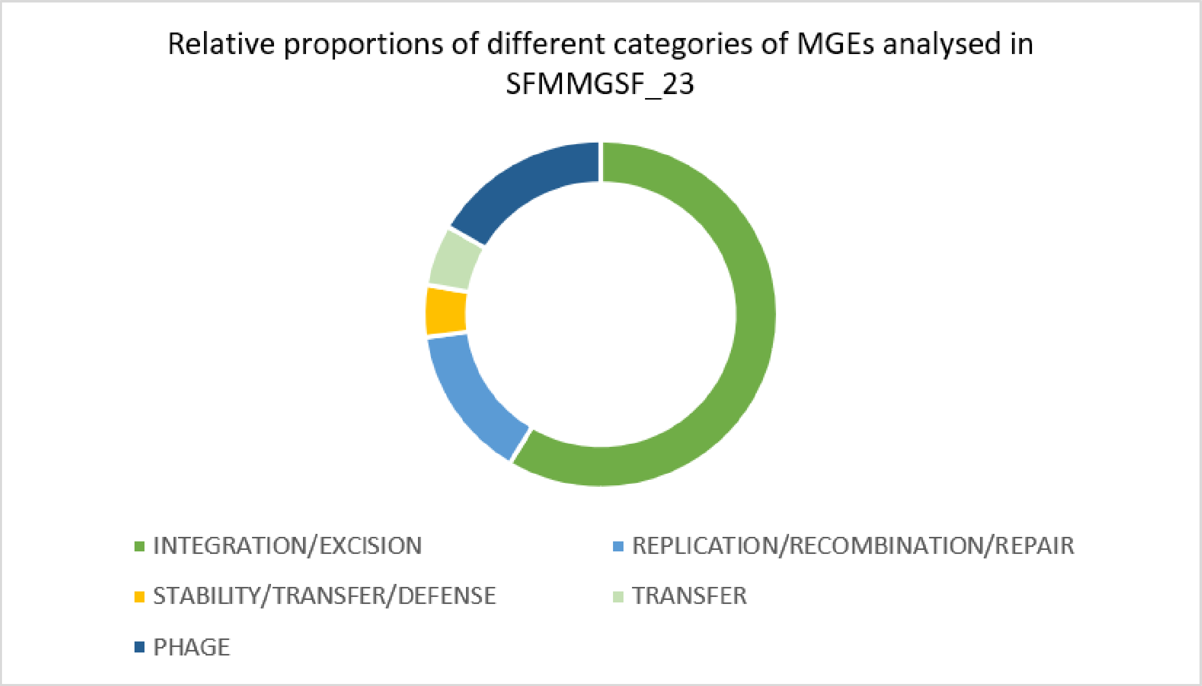

Supplement Fig. S3. Distribution of mobile genetic elements derived from phages, insertion sequences, integrative genomic elements (IGEs) and plasmids into different life cycle processes mediated by them.

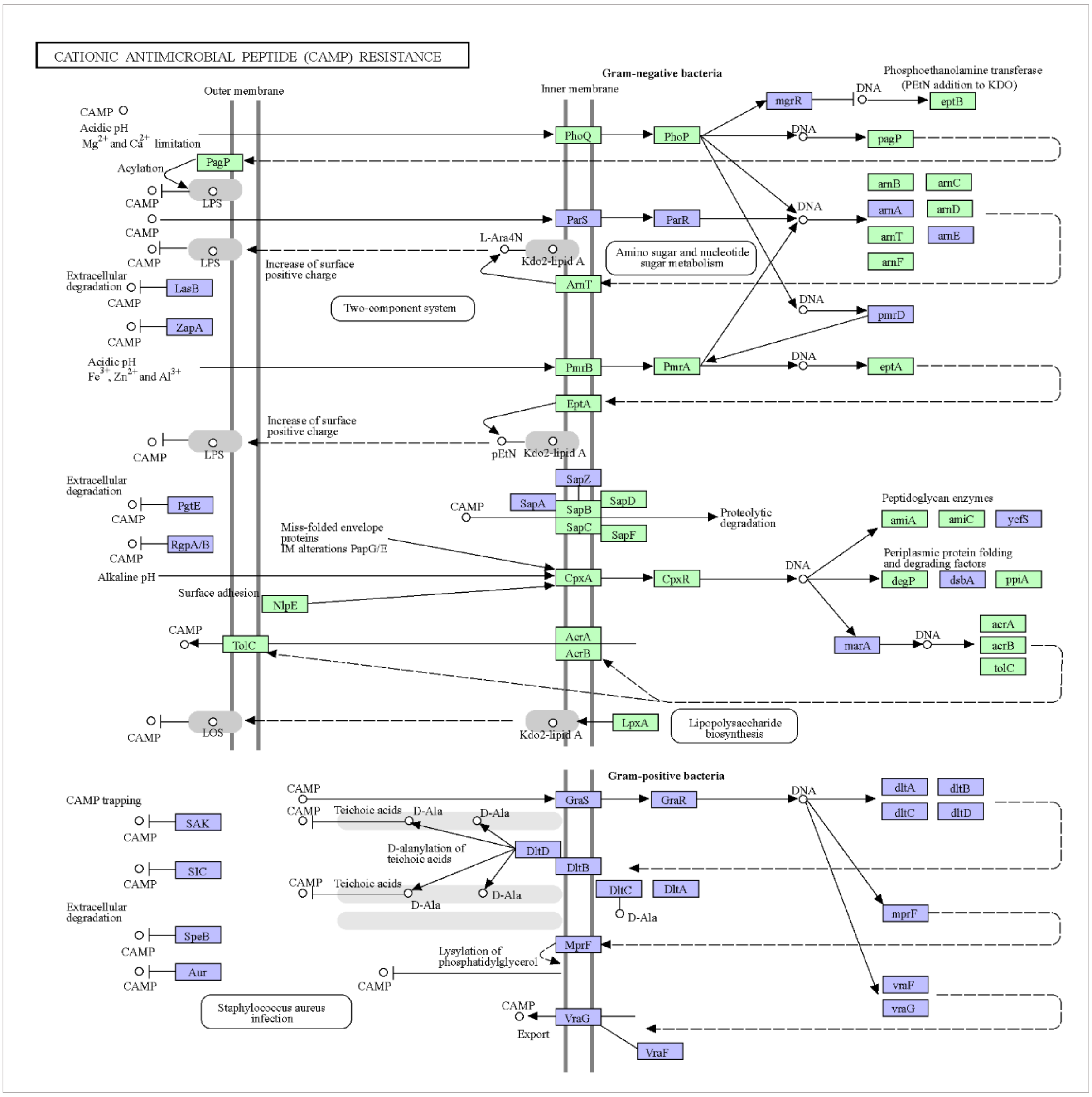

Supplement Fig. S4. Cationic antimicrobial peptide resistance pathway in Gram-negative bacteria (the identified resistance genes from SFMMGSG_23 is highlighted in green).

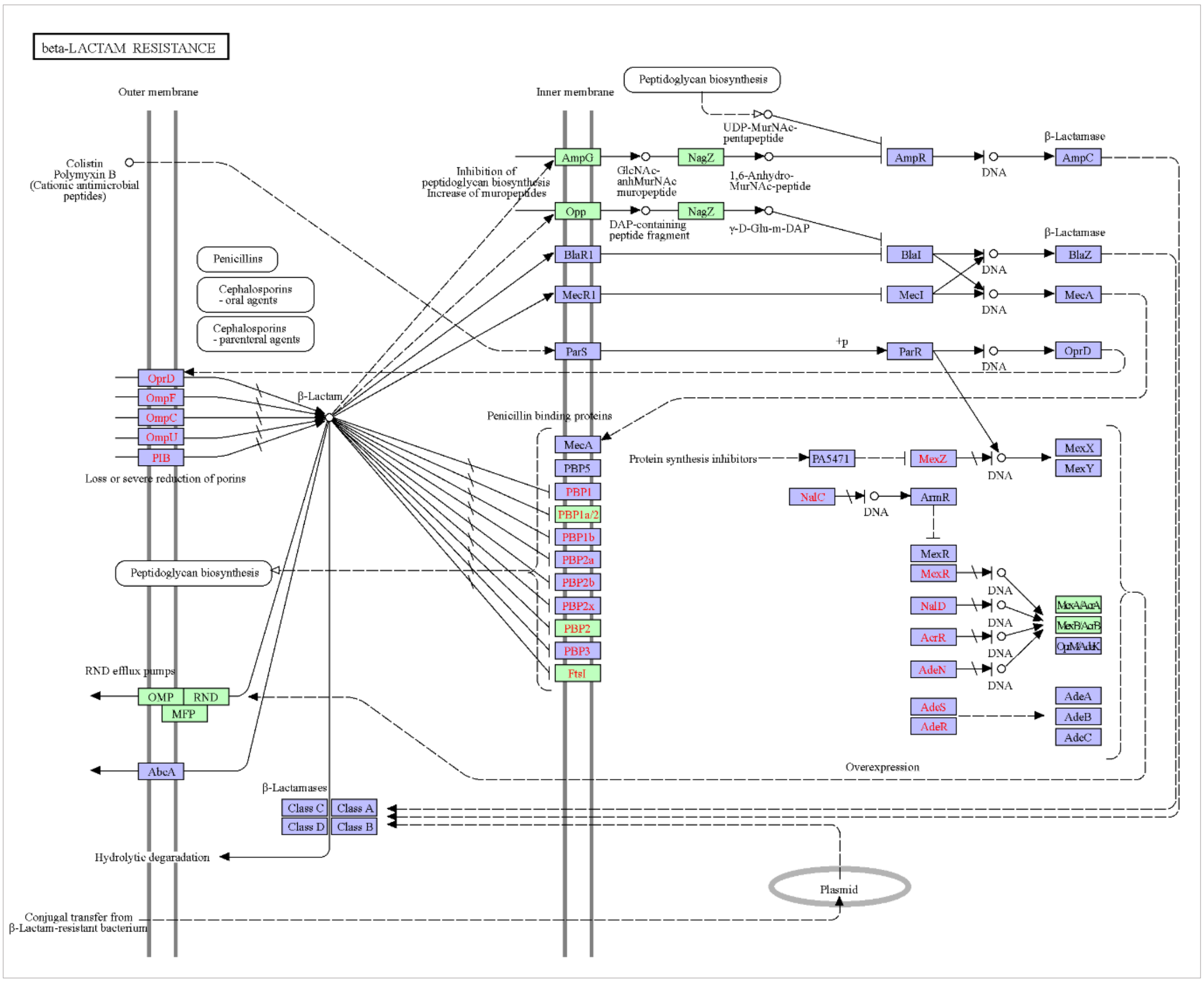

Supplement Fig. S5. Beta-lactam resistance pathway (the identified resistance genes from SFMMGSG_23 is highlighted in green).
